# Neurofeedback as a Treatment Intervention in ADHD: Current Evidence and Practice

**DOI:** 10.1007/s11920-019-1021-4

**Published:** 2019-05-28

**Authors:** Stefanie Enriquez-Geppert, Diede Smit, Miguel Garcia Pimenta, Martijn Arns

**Affiliations:** 1 Department of Clinical and Developmental Neuropsychology, Faculty of Behavioural and Social Sciences, University of Groningen, Grote Kruisstraat 2/1, 9712 TS Groningen, The Netherlands; 2 Department of Biomedical Sciences of Cells & Systems, Section of Cognitive Neuropsychiatry, University of Groningen, Groningen, The Netherlands; 3 Department of Experimental Psychology, Utrecht University, Utrecht, The Netherlands; 4 neuroCare Group, Munich, Germany; 5 Research Institute Brainclinics, Nijmegen, The Netherlands

**Keywords:** Neurofeedback, ADHD, Current status, Brain computer interface, Clinical practice

## Abstract

**Purpose of Review:**

Current traditional treatments for ADHD present serious limitations in terms of long-term maintenance of symptom remission and side effects. Here, we provide an overview of the rationale and scientific evidence of the efficacy of neurofeedback in regulating the brain functions in ADHD. We also review the institutional and professional regulation of clinical neurofeedback implementations.

**Recent Findings:**

Based on meta-analyses and (large multicenter) randomized controlled trials, three standard neurofeedback training protocols, namely theta/beta (TBR), sensori-motor rhythm (SMR), and slow cortical potential (SCP), turn out to be efficacious and specific. However, the practical implementation of neurofeedback as a clinical treatment is currently not regulated.

**Summary:**

We conclude that neurofeedback based on standard protocols in ADHD should be considered as a viable treatment alternative and suggest that further research is needed to understand how specific neurofeedback protocols work. Eventually, we emphasize the need for standard neurofeedback training for practitioners and binding standards for use in clinical practice.

## Introduction

Similar to many of his 9-year-old school peers, Brian was put on psychostimulants after complaints of poor concentration and impulsivity that met ADHD diagnostic criteria. Despite a remarkable improvement in his academic performance, parent and teachers noticed a reduction in appetite and weight loss after the onset of the medication. Moreover, when not under the effects of medication, inattention and impulsivity rebounded creating innumerous embarrassments to him and his family. His parents are now considering neurofeedback—a non-pharmacological and non-invasive intervention that has shown promising results in managing the ADHD symptoms in the long run and without side effects [1].

Despite being the most often applied and accepted treatments for ADHD, recent large-scale studies and meta-analyses have demonstrated limitations of psychostimulants and behavioral therapy. Thus, research and the development of non-pharmacological treatments such as neurofeedback have been recommended. To date, however, the clinical value of neurofeedback is still debated, with evaluations ranging from “efficacious and specific” [2, 3] to “fails to support neurofeedback as an effective treatment for ADHD”. [4•] In this contribution, we will introduce neurofeedback and review the application of neurofeedback to ADHD as well as its past and current evidence in the treatment of ADHD. We will also attempt to reconcile these seemingly discrepant research findings.

### Current Treatment Approaches in ADHD

Several guidelines exist for the diagnosis and treatment of children who have or are suspected of having ADHD. Among these are international, national, and various regional guidelines for general practitioners. Additionally, there are guidelines for youth aid and youth protective services.

Traditionally, the treatment of ADHD consists of pharmacotherapy, often complemented by behavioral therapy based on parent management training and mediation training for parents and teachers [5]. Additionally, classroom interventions, academic interventions, and peer-related interventions are being used as psychosocial therapeutic approaches [6]. Regarding pharmacotherapy, the administration of methylphenidate is often the method of choice (e.g., Ritalin, Concerta, Equasym, Medikinet); however, D-amphetamine, as well as non-psychostimulants, such as atomoxetine and guanfacine, are prescribed too [7]. Over the past years, the Multimodal Treatment Study of Children with ADHD and follow-up studies (the so-called MTA studies) have provided ample research regarding stimulant medication, behavioral treatments, their combination, and self-chosen community care. Results demonstrate that both stimulant medication and a combined treatment had a clear clinical benefit in the short term, but on the long-term group differences attenuate, as assessed after 24 months, as well as after 6 and 8 years [8]. These findings, in combination with studies indicating the potential side effects of pharmacotherapy [9•, 10], partial drug response [7], and the time and cost intensiveness of combining treatments due to the involvement of multiple professionals [6], have resulted in a growing interest into the development of alternative non-pharmacological treatments in ADHD.

For instance, computerized cognitive–based training approaches (e.g., working-memory and attention training) aim to reduce ADHD core symptoms and tackle neuropsychological functioning. Research into this topic is still in the early stages and more controlled studies regarding the effects on ADHD core symptoms are required [11]. Another alternative treatment method for ADHD which is already more extensively studied in the past is neurofeedback. In the following paragraphs, we will (i) introduce neurofeedback, (ii) present standard protocols for ADHD, (iii) review the past and current evidence in the treatment of ADHD, and (iv) depict the current status of institutional and professional regulation of the clinical implementation of neurofeedback.

### Definition, History, and Mechanism of Action of Neurofeedback

**Fig. 1:**
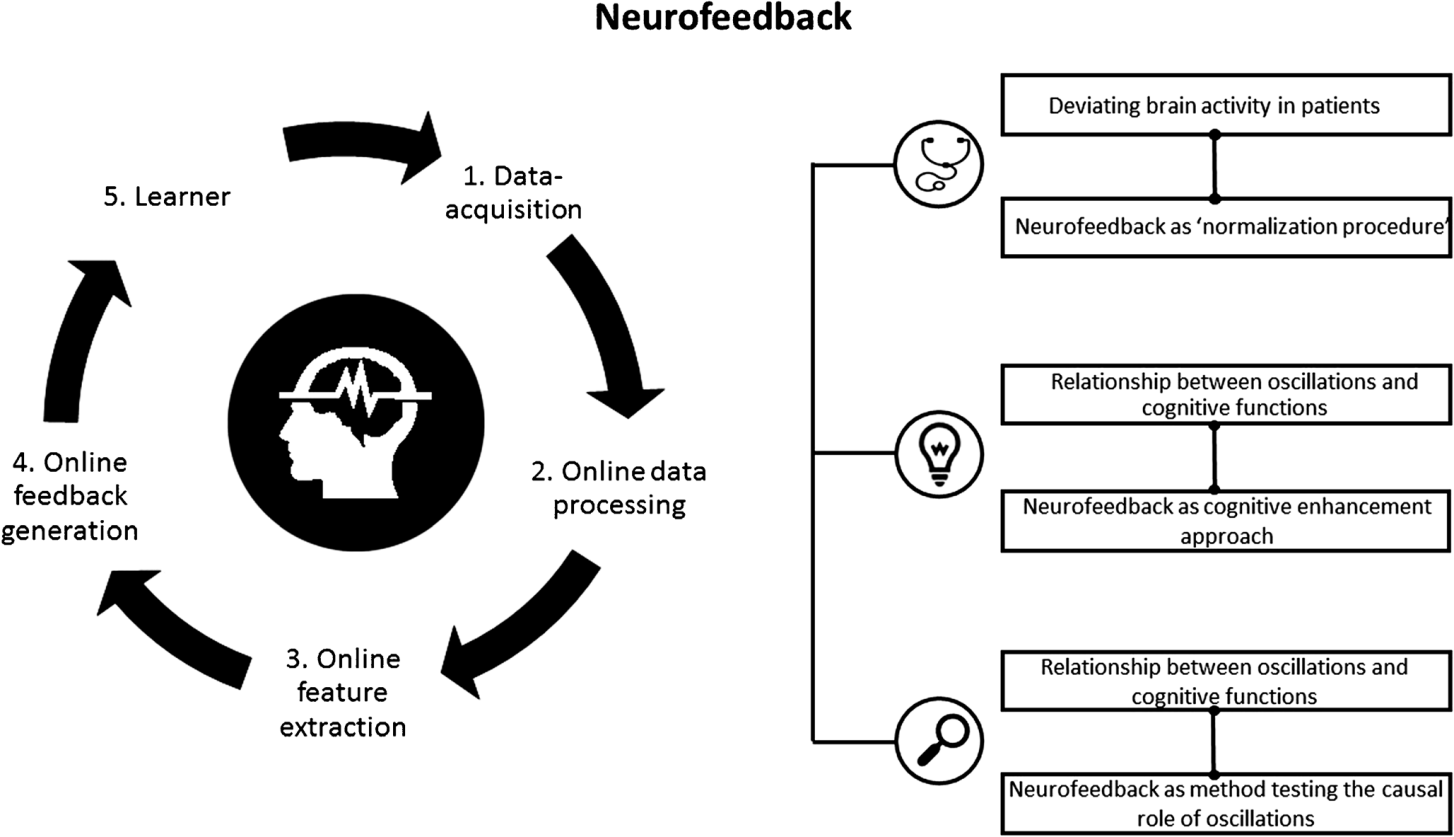
Overview neurofeedback: neurofeedback pipeline and three areas of neurofeedback application. The pipeline includes the five most important processing steps and parts of a neurofeedback system

Despite the recent popularity of neuromodulation techniques, neurofeedback is for the most part still an unknown territory. Neurofeedback is based on a brain-computer interface (BCI) and is implemented by a software system and a processing pipeline, altogether consisting of five elements (Fig. 1) [12•]. Neurofeedback measures the participant’s own brain activity, which is pre-processed (steps 1 and 2). Pre-selected brain parameters (a specific frequency band or a brain potential) are calculated online (step 3) and translated to signals that are fed back to the user in real time (step 4). Thus, selected features of brain activity are made perceivable for the participant. Through this feedback, the participant (step 5) can learn to self-regulate his own brain activity to directly alter the underlying neural mechanism of cognition and behavior.

It has been proposed that neurofeedback is based on principles of operant conditioning and procedural skills learning. Due to these learning mechanisms, neuroplasticity is expected to take place during neurofeedback training either via Hebbian plasticity or anti-Hebbian/homeostatic plasticity. Such intrinsic regulatory mechanisms are believed to prevent extreme states of brain activity, such as pathologically high or low synaptic strengths or oscillatory states; for further reading, see [13•].

Nowadays, neurofeedback is used in three ways: (i) as a therapeutic tool to normalize deviating brain activity and treat neurocognitive disorders, (ii) as a so-called peak performance training to enhance cognitive performance in healthy participants, and (iii) as an experimental method to investigate the causal role of neural oscillations in cognition and behavior. More precisely, the neurofeedback research is dominated by two streams: clinical research and neuroscientific inspired research, which is mainly based on recent methodological and technical innovations, as well as on an increasing knowledge about the neural correlates of behavior and cognition. Some examples of recently developed EEG neurofeedback protocols are the upregulation or downregulation of high alpha [14, 15], the upregulation of frontal beta [16], and frontal midline theta [17], but also neurofeedback protocols using fMRI neurofeedback [18•].

Historically, neurofeedback dates back to the initial discovery of the human electroencephalogram (EEG) by Hans Berger. Only 6 years after this breakthrough, two French researchers—Gustave Durup and Alfred Fessard—first reported that the EEG alpha rhythm could be subject to classical conditioning [19], which is thought to be one of the basic premises of neurofeedback. This initial observation was followed up by more systematic studies in the early 1940s that further demonstrated all of the Pavlovian types of conditioned responses could be demonstrated on the “EEG alpha blocking response”. [20] In a follow-up study, Jasper and Shagass [21] investigated further whether participants could also exert voluntary control over this alpha blocking response. In this study, they had participants press a button, which would switch the lights on and off, and use subvocal verbal commands when pressing the button, (e.g., “Block” when pressing the button and “Stop” when releasing the button). After five sessions, the subject was able to voluntarily suppress alpha activity, while the lights were off (a condition where normally synchronous alpha would be present). Despite these early developments, it was only in the 1970s that these same principles were applied more systematically, and the first clinical implications were described in the literature. These developments were motivated by the discovery of the anticonvulsant effects of sensori-motor rhythm (SMR) neurofeedback in cats [22] and subsequently humans [23]. The presumed role of SMR modulation on motor behavior was followed by the first demonstrations of the positive effects of SMR neurofeedback in hyperkinetic disorder [24]. Around the same 1960–1970 period, the first report of voluntary control over a slow brain potential called the contingent negative variation (CNV) or “bereitschaftspotential” (readiness potential, due to the property of this potential to emerge when preparing for action, e.g., when waiting in front of a traffic light) was reported [25], which laid the foundation of another well-known neurofeedback approach, namely of slow cortical potential (SCP) neurofeedback. The first application of SCP neurofeedback in ADHD was reported in 2004 [26]. The initial findings described above as SMR and TBR neurofeedback resulted into what we currently known as “frequency band neurofeedback.”

## Standard Protocols with ADHD

Theta/beta (4–7 Hz/12–21 Hz) ratio (TBR) neurofeedback strives to decrease theta and/or increase beta power in central and frontal locations. This protocol directly targets important electrophysiological characteristics such as high theta/beta ratios, high theta power, and/or low beta power commonly observed in children (for a review, see [27]) and adults with ADHD [28–30]. Recent randomized controlled trials suggest that 30 to 40 sessions of TBR neurofeedback were as effective as methylphenidate in reducing inattentive and hyperactivity symptoms and were even associated with superior post-treatment academic performance [31, 32]. It has been proposed that the effects of TBR neurofeedback on ADHD might be explained by the learned self-regulation of attention [33] as evidenced by enhanced amplitude of endogenous evoked-related potentials such as the P300 [34]. However, more neuroscientific evidence is needed to determine the specific mechanisms by which TBR neurofeedback might impact cognitive functioning in ADHD.

SMR neurofeedback training over the sensori-motor strip (predominantly in the central right hemispheric region) was first applied to ADHD children by Lubar and colleagues [24, 35], based on the functional association of the sensori-motor rhythm with behavioral inhibition and the promising results in reducing cortical excitability in epileptics obtained by Sterman, MacDonald, and Stone [36]. Lubar’s seminal studies revealed that the beneficial hyperactivity-reducing effects of a combined SMR/theta neurofeedback training were maintained after psychostimulants was withdrawn in hyperactive children.

Studies suggest that SMR neurofeedback training reduces inattentive and hyperactive/impulsive symptoms in ADHD children to the same extent as TBR training and comparable number of treatment sessions. However, the two protocols might achieve the same results through distinct mechanisms. Arns, Feddema, and Kenemans [37] provided evidence that ADHD patients trained with the SMR protocol showed decreased sleep onset latency (SOL) and improved sleep quality in comparison to those administered with TBR, midway treatment. A mediation analysis revealed that this normalized sleep mid-treatment was responsible for the improved inattention post-treatment. The improvements in ADHD symptoms following SMR training might hence be the result of the vigilance stabilization mediated by the regulation of the locus coeruleus noradrenergic system of which activation has been shown to impact the sleep spindle circuitry [38]. This explanation seems to be in line with previous indications that patients with ADHD present delays in SOL [39] and that SMR training increases sleep spindle density and improves sleep quality in healthy adults [40].

Another standard protocol is the self-regulation of SCP [41, 42••] after around 35 sessions. SCP neurofeedback is based on the learned self-regulation of cortical activation and inhibition which are associated with the electrical negativation and positivation of slow cortical electrical deflections respectively. These periodical shifts from electrical positivity to negativity have been described as a phasic tuning mechanism in the regulation of attention [43] as shown by the enhanced reaction time, stimulus detection, and short-term memory during the negative shift phase [44]. Since SCP, of which the CNV is an example, are closely associated with preparatory motor responses with a maximal topographic representation in the motor areas, the vertex is usually the site of choice for training. Differently from TBR and SMR protocols which are typically unidirectional (i.e., instructions either require the participant to increase or decrease the power of the EEG parameter), the self-regulation of SCP usually involves the training in generating both cortical activation and inhibition. In the case of ADHD, the therapeutic focus is on promoting an increase in the firing probabilities of the underlying cortical areas (i.e., negativation). Another difference relative to frequency neurofeedback is that in SCP neurofeedback the learning trials are higher in number and considerably shorter in duration. Interestingly, it has been hypothesized that SCP might also be associated with improvements in sleep. The generation of slow oscillations, in particular negative slow direct current, shifts training during SCP neurofeedback, might exert control over the sleep spindle circuit and therefore facilitate the transition from wakefulness to sleep [45].

## Current Status of Efficacy of Standard Protocols for Neurofeedback in ADHD

As with any emerging new treatments, knowledge of technical aspects of the treatment, proper standards, and education are crucial for appropriately evaluating the merits and pitfalls of neurofeedback. Unfortunately, the unfounded assumption that “neurofeedback = neurofeedback” is often made. Neurofeedback can differentially impact brain functioning depending on the kind protocol and implementation the same way as different pharmacological treatments do (e.g., antidepressants and analgesic drugs). As an illustration, neurofeedback treatments such as the earlier mentioned SMR, TBR, and SCP neurofeedback are well-investigated and effective in the treatment of ADHD while other approaches such as posterior alpha enhancement have been found to be not effective (for a review, see [3].

Especially when restricted to standard protocols such as TBR, SMR, and SCP protocols [3], neurofeedback is a well-investigated treatment for ADHD. This has become evident from several meta-analyses [2, 46••, 47], including a critical meta-analysis from the European ADHD Guidelines Group (EAGG) that also conducted a sensitivity analysis focused on so called “blinded” ratings (i.e., teacher reports only) [4•]. Blinded ratings have usually lower effects sizes than ratings by people most-proximal to the child and therefore least blinded (e.g., parents) and both rating types are only modestly correlated [48]. One explanation for this may be that the rating types focus on different aspects of ADHD symptoms. This is reflected in studies showing different rating-ADHD aspect associations, as for instance parent ratings of hyperactive-impulsive behaviors were found to be correlated with genetics [49], whereas teacher ratings have been shown to be associated to medication effects [50], most likely due to the fast onset of action of psychostimulants. To come back to the latter meta-analysis [4•], the researchers did not find an effect of neurofeedback in general on teacher-rated ADHD symptoms, but there was an effect when the analysis was restricted to the above mentioned “standard protocols.” Finally, a recent meta-analysis that included 10 RCTs and specifically looked at long-term effects of neurofeedback, compared to active treatments (including psychostimulants) and semi-active treatments (e.g., cognitive training), found that after on average 6 months follow-up, the effects of neurofeedback were superior to semi-active control groups and no different from active treatments including methylphenidate [46••]. Interestingly, this meta-analysis confirmed the trend for medication effects to diminish with time, and the effects of neurofeedback—without additional sessions being conducted—to increase with time. These data suggest the promising aspect, namely of long-term efficacy, of neurofeedback. Currently, one of the largest and most comprehensive double-blind multisite RCT is carried out: the International Collaborative ADHD Neurofeedback study (ICAN). This study consists of a cross-site investigation team with different background of ADHD treatment approaches assessing 140 participants in total (see the study design in [51]), and results are foreseen to be published in 2019.

## Current Status of Institutional and Professional Regulation of Clinical Neurofeedback Implementations

Although standard protocols turn out to be efficacious and specific, the practical implementation of neurofeedback as a clinical therapy is currently not regulated. This applies to the educational standards, medical security, and the usage of standard protocols indicated for specific disorders such as ADHD. The lack of regulation and agreed upon standards comes with the danger of patients being treated with ineffective neurofeedback protocols applied by unlicensed personal (or even worse by people without any health-related background). For instance, although practitioners should stick to standard protocols with functional specificity of the frequency and topographic locations, clinical practice often deviates from what is recommended by research. The lack of regulation and missing standards have furthermore caused a surge in commercial driven applications and proclaimed “innovations” of neurofeedback protocols and implementations. Several studies have now demonstrated that some of those “innovations” and implementations do not work. One example of such ineffective technique is the SmartBrain neurofeedback approach using the “NASA patented engagement index” with Sony PlayStation feedback [51, 52]. Additionally, there is no evidence in favor of the efficacy of unconventional neurofeedback protocols used in some neurofeedback clinics [53] and frequently advertised applications such as *Z* score and LORETA neurofeedback [54]. Unfortunately, these proclaimed innovations and commercial-driven applications only add noise to the ongoing debate of neurofeedback efficacy and risk “throwing the baby out with the bathwater.” However, above all this demonstrates the need for further research into the effectiveness of already available and newly developed neurofeedback protocols (i.e., the number of sessions, targeted brain area, selected brain parameter, working mechanism) in addition to proper “agreed-upon standards” and training within the field of neurofeedback.

Neurofeedback researchers and practitioners can affiliate to scientific and professional organizations at the international and national level. On an international level, there are mainly two societies. The Society of Applied Neuroscience (SAN) (http://www.applied-neuroscience.org/) is an EU-based nonprofit membership organization for the advancement of neuroscientific knowledge and development of innovative applications for optimizing brain functioning (such as neurofeedback with EEG, fMRI, NIRS). The International Society for Neurofeedback & Research (ISNR (https://www.isnr.org) is a membership organization aimed at supporting scientific research in applied neurosciences, promoting education in the field of neurofeedback, albeit not always clearly separating commercial and objective interests. Other neurofeedback societies or organizations are often connected to certain neurofeedback equipment manufacturers and have (seemingly) conflicting interests. Furthermore, the Biofeedback Certification International Alliance (BCIA) is a broader international licensure also including biofeedback (www.BCIA.org).

## Conclusions

Recent years witness a renewed interest in neurofeedback in response to the lack of long-term effects for both medication and behavioral therapy and the side effects of medication. Herein, we provide evidence for the efficacy and specificity of standard neurofeedback protocols, namely theta/beta, sensori-motor rhythm, and slow cortical potential. In line with the guidelines for rating evidence developed by the APA, “standard” neurofeedback protocols have been considered to be “Efficacious and Specific, Level V” in the treatment of ADHD (AAPB Guidelines: [57]).

However, currently there are no uniform standards regarding training courses for neurofeedback that are accepted by expert associations, neither national-wide, nor in the EU or USA. While performing neurofeedback in a therapeutic context, a thorough basic training, a distinct technical understanding of the medical devices, the software, and the EEG caps, as well as continuing education, are imperative. Regarding the medical security performing neurofeedback in a clinical context, neurofeedback devices (hardware: amplifier and EEG caps, neurofeedback software) are neither regulated in a strict way. However, it is essential that besides the absolute minimum technical requirements after the Medical Device Regulation (MDR) EU 2017/745), neurofeedback devices should be regulated by both the CE (that confirms a medical device meets the essential MDR requirements) and a European equivalent of the Food and Drug Administration (FDA). The FDA enforces laws to protect the consumer’s health, safety, and pocketbook. Such potential regulating mechanisms could be implemented by the European medicine regulatory network. In short, tasks ahead concern regulating neurofeedback as therapy, developing internationally accepted binding standards for education and NF implementation and the qualification of neurofeedback trainers.

Last but not least, Brian—now 4 years later—discontinued his medication successfully under medical supervision. Due to neurofeedback, his impulsivity symptoms strongly reduced and he gained control over his concentration, doing well in high school performance.
